# AAPM Medical Physics Practice Guideline 5.a.: Commissioning and QA of Treatment Planning Dose Calculations — Megavoltage Photon and Electron Beams

**DOI:** 10.1120/jacmp.v17i1.6166

**Published:** 2016-01-08

## ORIGINAL CITATION

Jennifer B. Smilowitz, Chair, Indra J. Das, Vladimir Feygelman, Benedick A. Fraass, Stephen F. Kry, Ingrid R. Marshall, Dimitris N. Mihailidis, Zoubir Ouhib, Timothy Ritter, Michael G. Snyder, Lynne Fairobent, AAPM Staff. AAPM Medical Physics Practice Guideline 5.a.: Commissioning and QA of Treatment Planning Dose Calculations — Megavoltage Photon and Electron Beams. J Appl Clin Med Phys. 2015;16(5).

Author description of the error and correction: One author, Mark Geurts, was inadvertently omitted. The correct author list should read:

Jennifer B. Smilowitz, Chair, Indra J. Das, Vladimir Feygelman,

Benedick A. Fraass, Mark Geurts, Stephen F. Kry, Ingrid R. Marshall, Dimitris N. Mihailidis, Zoubir Ouhib, Timothy Ritter, Michael G. Snyder, Lynne Fairobent, AAPM Staff.

